# Supplementary figures and images for: Iliski, a software for robust calculation of transfer functions
Source: PLoS Comput Biol. 2021 Jun 14;17(6):e1008614. doi: 10.1371/journal.pcbi.1008614 (PMC8224889; doi:10.1371/journal.pcbi.1008614)

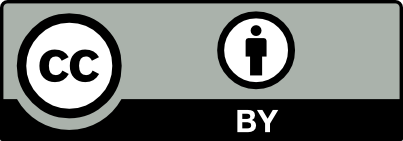

Supplement: S2 Data — Current version is available at https://github.com/alike-aydin/Iliski. (ZIP) [file pcbi.1008614.s002.zip › Iliski-1.0.0/LICENSE.png]
