# Supplementary material for: Iliski, a software for robust calculation of transfer functions
Source: PLoS Comput Biol. 2021 Jun 14;17(6):e1008614. doi: 10.1371/journal.pcbi.1008614 (PMC8224889; doi:10.1371/journal.pcbi.1008614)
Supplement: S1 Text — Current version is available in the GitHub repository at https://github.com/alike-aydin/Iliski. (PDF) [file pcbi.1008614.s004.pdf]

Iliski: User Manual

Ali-Kemal Aydin

INSERM, CNRS, Institut de la Vision, Sorbonne Université, Paris, France

Davide Boido

NeuroSpin, Bât 145, Commissariat à l'Energie Atomique-Saclay Center, 91191, Gif-sur-Yvette, France

Iliski is a software developed in the Charpak Team. It aims to compute Transfer functions between any two timeseries, to represent their dynamic relationship. *Iliski* is further described in the following pre-print available on BioRxiv.

Contents

Iliski: User Manual

Ali-Kemal Aydin & Davide Boido **1**

1 Overview of *Iliski* 4

1.1 Where to find *Iliski*? 4

1.2 Overview of the workflow 5

1.3 How to use *Iliski*? 5

2 Input data 5

2.1 Input files 5

2.2 Use a boxcar function as From signal 9

3 TF computation process 10

3.1 Data pre-treatment 10

3.2 Transfer Function computation 12

3.3 Evaluation of the TF 17

3.4 Available options 18

4 Saving a computation and accessing a previous one 19

4.1 Saving as a MAT-File 19

4.2 Saving as Excel file 20

5 *Iliski* as a suite of scripts 20

6 Specifications 20

7 Contact 20

Glossary 20

Acronyms 21

Bibliography 21

1 Overview of *Iliski*

Iliski ([ɪlɪʃkɪ]) means *relationship* in turkish. It computes relationships between timeseries[1]of any type. Figure 1 shows the software's Graphical User Interface (GUI). The user can also use the scripts independently of the GUI. *Iliski* is written with MATLAB.

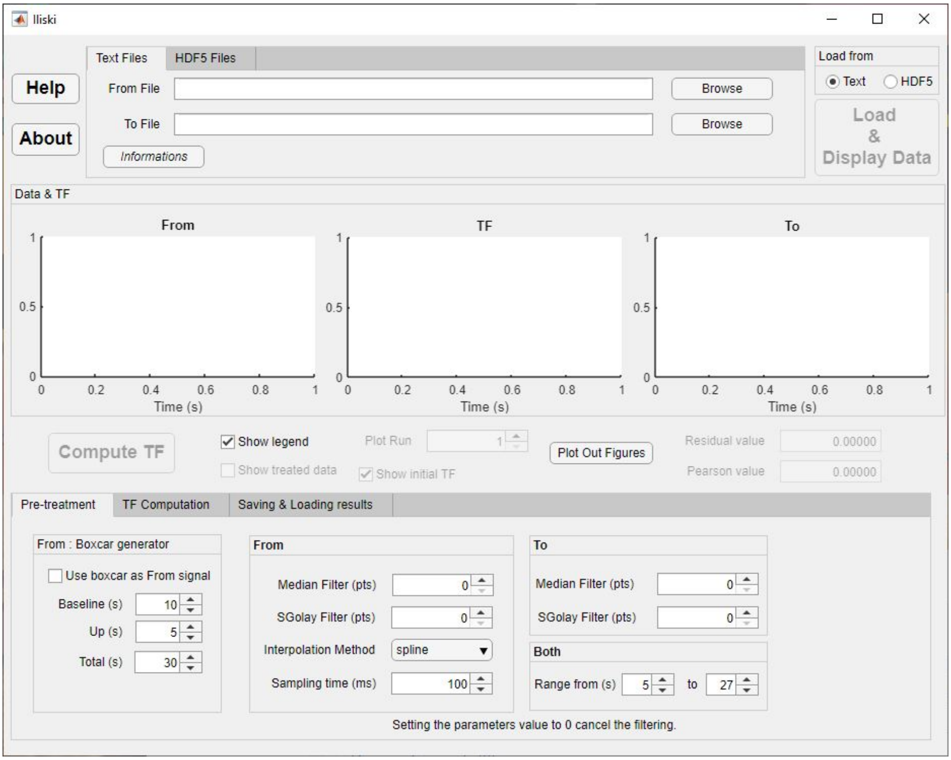

Figure 1: View of *Iliski* right after it being launched.

1.1 Where to find *Iliski*?

Iliski can be downloaded on GitHub at the following link: https://github.com/alike-aydin/Iliski. Refer to the `ReadMe` part of the repository for more details on what version to download depending on your system and MATLAB installation.

[1]Although we state here and in *Iliski* that data are time-dependent, there are no such requirement. One can use any type of x coordinates, but will have to be careful with the options regarding the time to keep the resulting TFs reliable.

Some example datasets are available on Zenodo: https://doi.org/10.5281/zenodo.3773863 and some are already in the `ExampleData` folder.

1.2 Overview of the workflow

Iliski gets two different signals as inputs: the 'From' signal, herein after refered as From, and the 'To' signal, similarly refered as To, both functions of time[1]. *Iliski* will compute Transfer function (TF) to accurately solve (1.1). The $*$ operator denotes the convolution operation.

$$From(t) * TF(t) = To(t) \tag{1.1}$$

Each option of *Iliski* aims at modifying the way the TF will be computed, either directly with the computation options or indirectly with the signal pre-processing options. There are three main parts to the computation process, illustrated in Figure 2:

1. Data pre-treatment: From and To data are modified to allow for a better TF computation.
2. Transfer function computation: there are several ways to compute a TF.
3. TF evaluation: a TF computed from a parametric function optimization algorithm never perfectly predicts the test dataset. It has to be evaluated.

Every option of each part is described in the section 3.2 (page 12).

1.3 How to use *Iliski*?

As written above, *Iliski* can be used in two different ways: through its GUI, or by directly calling its functions in one's own scripts. Note that everything has been written in MATLAB 2018a (see section 6, page 20). The GUI version can be used as a MATLAB file, thus needing an operational MATLAB installation, or as a standalone application. Refer to the section 5 (page 20) for more details on the various functions at the user's disposal for using *Iliski* as a suite of scripts.

2 Input data

2.1 Input files

Iliski can treat various file formats as input data. The simpler one is text-based, e.g. `.txt`, numerical data-file. *Iliski* also allows for HDF5 files but it will need

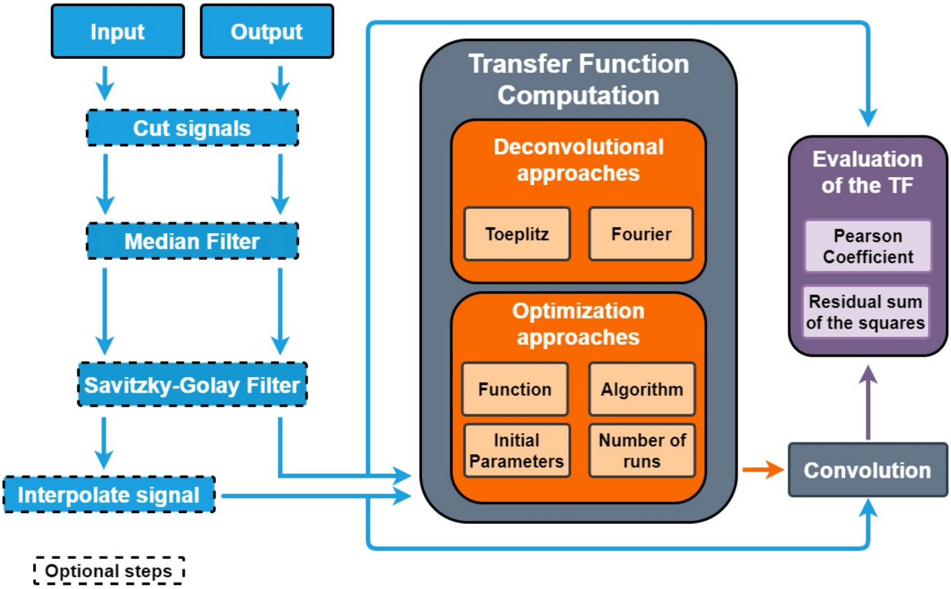

Figure 2: *Iliski*'s workflow is separated in three main parts, from left to right: pre-treatment of the input data, computation of the TF and evaluation of the computed TF.

a more restrictive format, as described in section 2.1.2.1 (page 7). There are two tabs, depending on the file format. The user must also specify the format correspondant to the input data above the **Load and Display** button (see Figure 3 and Figure 4). Note that Iliski was not coded to handle complex-valued signals.

Iliski supposes From and To data are timeseries. For this reason, we will call 'time' the vector of x coordinates of From and To. As such, it needs to have a time vector matching the data vector for both. Note that the sampling does not need to be identical between both signals: *Iliski* provides interpolation to account for that (for more details see section 3, page 10).

2.1.1 Text-based files

Both From and To must have their own file (see Figure 3). Data inside a text-based file should be in a column form, separated either by tabulations or spaces. Depending on the number of columns, *Iliski* will interpret the files differently:

- Two-columns file: the first column is the time vector, while the second one is the data vector.

- One-column file: the only column will be used as the data vector. The x vector will be artifically created as a vector starting from 0 up to the number of points, with a step of 1.
  Make sure that this interpretation will not create discrepancies between the From and To signals.

- More than two columns file: only the first two columns will be accounted for, respectively as time and data vectors.

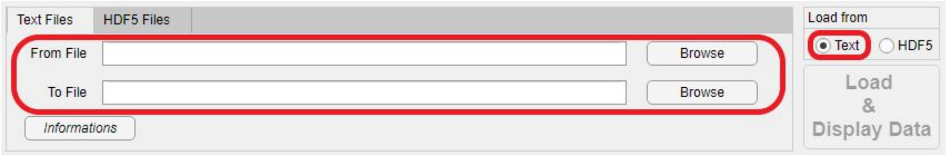

Figure 3: Paths to the From and To text data files should be put in the left red box. The right red box specifies what format is to be loaded.

2.1.2 HDF5 files

Iliski accesses data stored inside HDF5 file (Hierarchical Data Format)[2]. Paths to the HDF5 files containing the From and To data must be written in the boxes circled in Figure 4. Note that if the HDF5 file containing the From and To data are the same, a checkbox is placed on the right to avoid typing twice the same path. Figure 5 shows *Iliski* after loading data from an HDF5 file (`VT31.h5`, available on the GitLab repository).

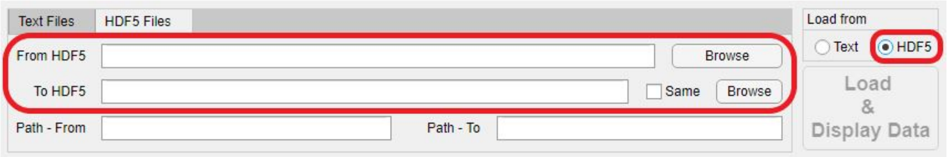

Figure 4: Paths to the From and To HDF5 data files should be put in the left red box. The right red box specifies what format is to be loaded.

2.1.2.1 HDF5 files requirements for *Iliski*

HDF5 files contains data organized in groups, i.e. folders and subfolders. To access the data to compute a TF on, *Iliski* has two separate text boxes for the

[2] HDF official website & HDF Wikipedia page

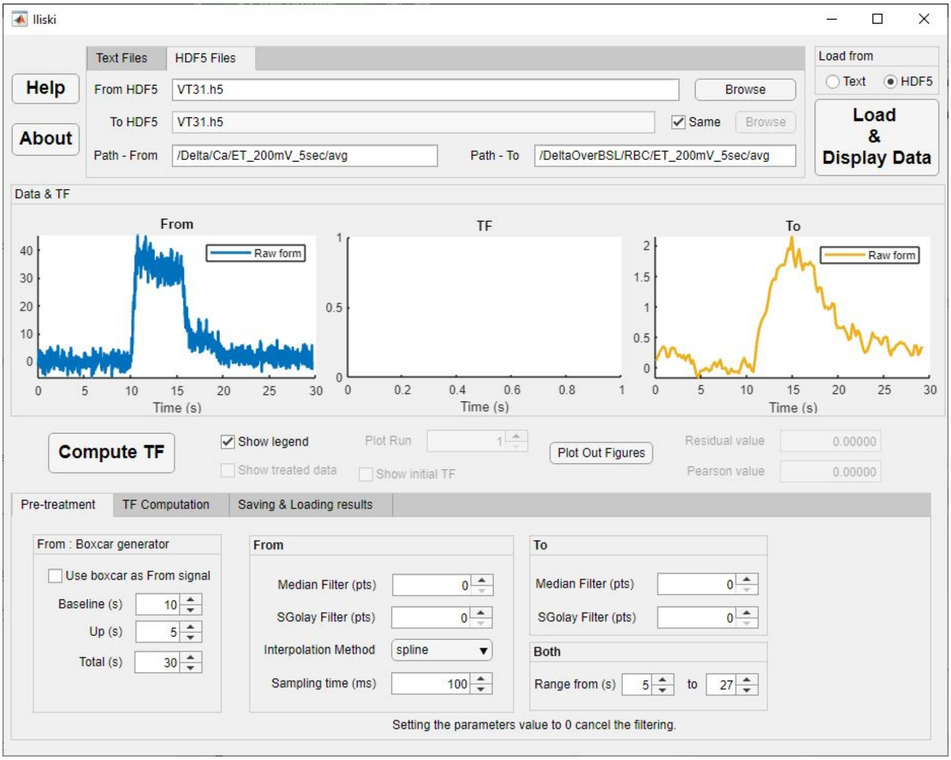

Figure 5: From and To data are loaded from an HDF5 file.

From and To data files. Figure 6 shows where they are.

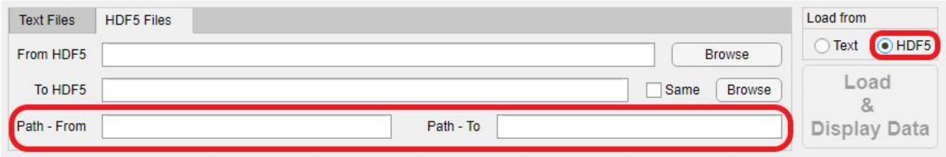

Figure 6: Paths to the From and To data, inside the HDF5 files, should be put in the boxes circled in red.

For *Iliski* to retrieve the corresponding time vector inside the HDF5 arborescence, it follows two rules (see Figure 7):

- The time vector is supposed to have the same dimensions as the data vector, since every datapoint has its corresponding time point.

- The time vector is placed in the same group as the data vector and is named ’*time*’.

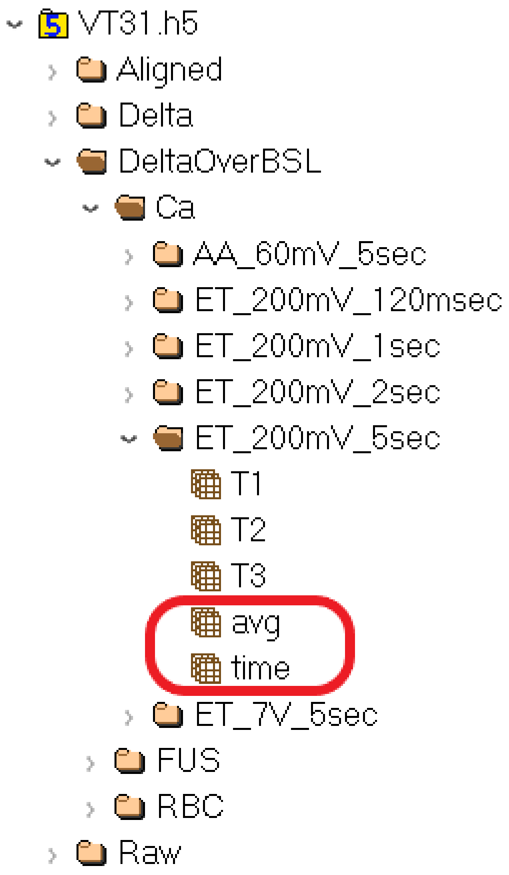

Figure 7: The x coordinates vector must be named ’*time*’ and be in the same HDF5 group as the y coordinates vector, here ’*avg*’ for instance. Screenshot from the HDFView software.

2.2 Use a boxcar function as From signal

We developed *Iliski* to study the neurovascular coupling. One main application is a better understanding of the Blood Oxygen Dependent Level based Functional Magnetic Resonance Imaging (BOLD-fMRI). Extracting activated brain ar-

eas from BOLD-fMRI requires to somehow represent the neural activation. If no information is available on the neuronal activity, a conservative approach consists in using the temporal representation of the stimulus application with the shape of a **boxcar function** (see Figure 8) going from 0 to 1 when the subject under the BOLD-fMRI is stimulated[3].

Iliski lets the user use a **boxcar function** as the From signal if needed. Figure 8 shows where the parameters are placed in the GUI:

- **Baseline**: Duration, in second, of the pre-stimulus 0 period.
- **Up**: Duration, in second, of the stimulus 1 period.
- **Total**: Duration, in second, of the total timeserie (baseline + stimulus + post-stimulus periods).
- **Sampling time**: Time between points, in milliseconds.

Once the corresponding boxes are filled with the paths to the input files, a click on **Load and Display Data** makes *Iliski* load them. If you want to use a boxcar function, fill the To path(s) before checking the checkbox among the boxcar parameters.

3 TF computation process

Figure 2 outlines the main parts of the computation process.

Once *Iliski* loads your data, as described in section 2 (page 5), the computation process can start. After clicking on the **Compute TF** button in the GUI, *Iliski* will first treat your data.

3.1 Data pre-treatment

Iliski allows for **cutting signals to a given time range**, specified by two parameters: start and end timepoints. Both of them are in the same unity as your time vector, which is displayed as ”seconds” in the GUI.

Your input data, either From or To, may be noisy. To get a meaningful TF, i.e. a meaningful relationship between your input data, removing part of the noise can be useful while paying attention to keep as much information as necessary in your timeseries. *Iliski* offers two methods you may combine:

1. **Median Filter**[4]: to remove outliers from your timeseries. The parameters

[3] Dear BOLD-fMRI people, apologies for the quick-and-dirty explanation.
[4] Moving median function *movmedian* MATLAB documentation

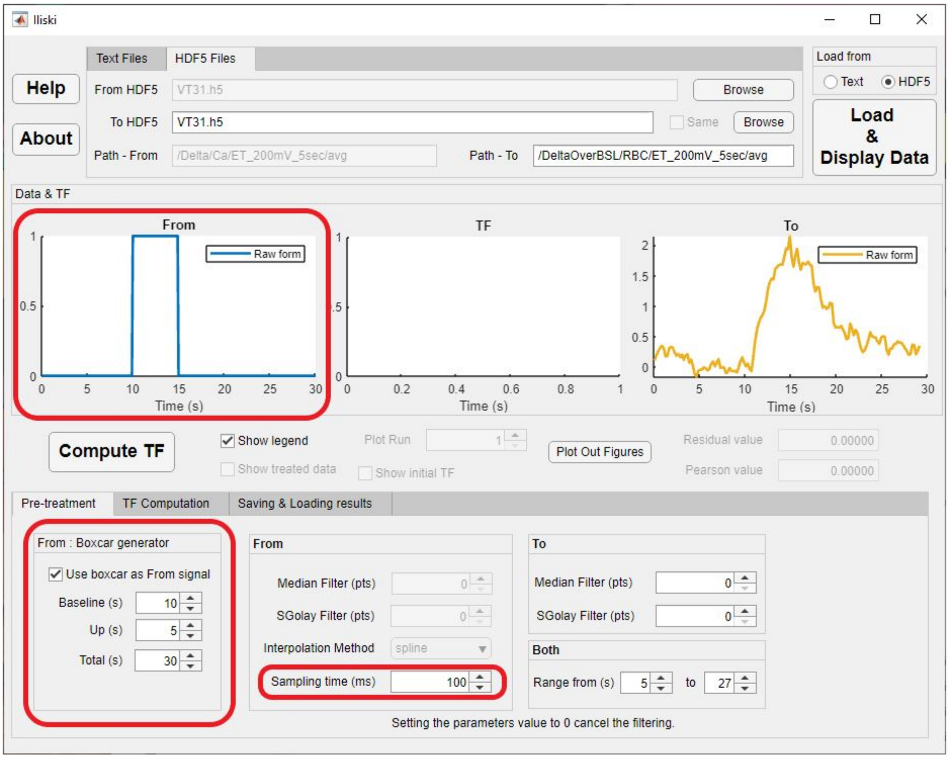

Figure 8: The lower red circle outlines the parameters placement for the boxcar function, while the upper red circle show the resulting signal.

value is the number of points to apply the median filter on. If selected, this filter is applied first.

2. **Savitzky-Golay Filter**[5]: to smooth your timeseries while avoiding the shift caused by a sliding average. In practice, he filter will go over each point of the timeserie and fit a 3rd degree polynomial. The current point will then be replaced by the time point corresponding in the fit. The parameter corresponds to the number of points to fit, note that it has to be an **odd number** so that the current point is the center of the fit.

Both these parameters are available for the From and To signals. Use 0 as parameter to specify not to use these filters.

The last step of the data pre-treatment is the **interpolation** of the input data.

[5] Savitzky-Golay filter function *sgolayfilt* MATLAB documentation

Iliski has different behaviors depending on the TF computation you chose (see section 3.2, page 12):

- **Deconvolutional approaches**: either through **Fourier Transform** or **Toeplitz Matrix.** Both signals must share the same time vector, thus, both From and To have to be interpolated to the same sampling time.
- **Optimization algorithm**: either with a **deterministic** or **non-deterministic algorithm**. This TF computation process does not require the To to have the same sampling time as From (see section 3.3, page 17).
- Using a **boxcar function** as From signal: interpolation is not performed to avoid changing the shape of the boxcar function (note that in Figure 8, the pre-treatment parameters are grayed).

There are two parameters to set for the interpolation:

- **Interpolation method**[6]: *Iliski* uses the *interp1* MATLAB function. The drop-down list allows you to chose the interpolation method used by the *interp1* function.
- **Sampling time**: the new sampling time in milliseconds (namely one thousandth of the unity of your time vector[7]). Note that if your time vector is regularly spaced and you put the original sampling time, no interpolation will occur. Also, note that this parameter should be **equal to the sampling time of the boxcar function** if you use it as From timeserie, otherwise an error message will pop-up.

See Figure 9 to get the parameters placement on the GUI.

3.2 Transfer Function computation

Once the input data is pre-treated, the TF can be computed. *Iliski* offers several ways to do so. See the attached scientific paper for more details on the difference between them (BioRxiv paper). The parameters for each type are described in the subsection 3.2.3 (page, 16).

[6] Interpolation function *interp1* MATLAB documentation
[7] Well, not necessarily time as already stated page 4

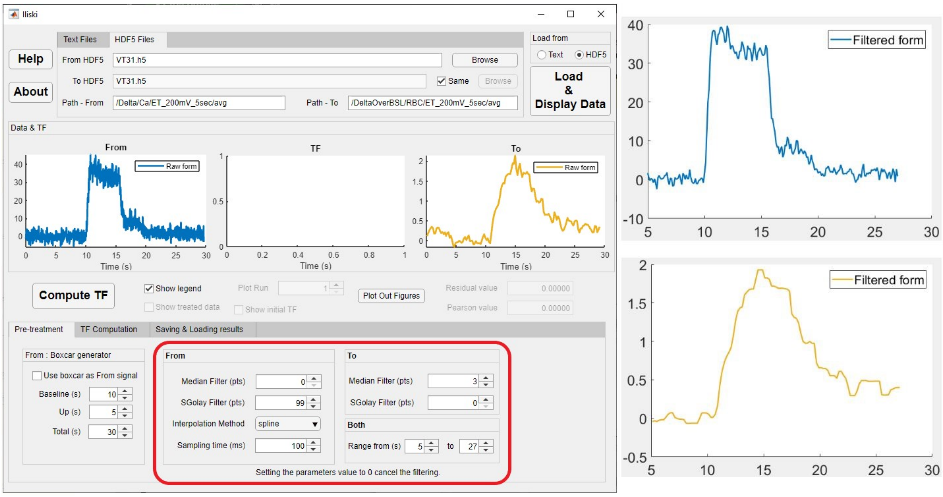

Figure 9: Circled in red are the parameters for the pre-tratment of the input data. On the right are the resulting data, treated according to the parameters on the left.

3.2.1 Deconvolutional methods

Deconvolutional methods are the canonical way to compute TFs. They avoid making any assumptions on the shape of the relationship. However, they are very sensitive to the noise of the signal: although there is a pre-treatment step, it may not be enough to get a clean enough signal while keeping all the relevant information.

Iliski proposes two deconvolutional methods:

- **Fourier Transform**
- **Toeplitz Matrix**

Most of the time, they will both give close results.

3.2.2 Optimization algorithms

Optimization algorithms require to define a function whose parameters are to be optimized to minimize a cost function value (see section 3.3, 17). *Iliski* has pre-registered functions, useful in the neurovascular coupling field, but one can use his own function too (see section 3.2.2.3, page 14). We propose two classes of optimization algorithms in our software: deterministic and non-deterministic algorithms.

3.2.2.1 Deterministic algorithms

3 different deterministic algorithms are proposed:

- **fminsearch**[8]: unconstrainable ;
- **fminunc**[9]: unconstrainable ;
- **fmincon**[10]: constrainable.

For **fmincon** and the non-deterministic algorithms, constraints can be applied to the optimization of the parameters. The user can define upper and lower bounds for each parameters in the GUI (see Figure 10).

3.2.2.2 Non-deterministic algorithms

Non-deterministic algorithms have a random component in their optimization process, to be able to jump from one cost-function minimum to another. Their purpose is to skip local minima and to reach the global minimum. In *Iliski*, we propose:

1. **Simulated Annealing**[11]: constrainable ;
2. **Simulated Annealing** followed by **fminunc**: the point here is to try to get a deeper minimum by adding a layer of determinictic optimization over the non-deterministic one. The underlying hypothesis is that Simulated Annealing may not get to the absolute minimum but still be inside the well, in which case **fminunc** will go down to the minimum of that well.

3.2.2.3 Pre-registered and personalized functions

Iliski has two embedded functions, with t being the time and p the parameters to optimize:

- **1-Gamma Hemodynamic Response Function**: with H being the Heaviside function and $Gamma$ the Gamma function.

$$H(t - p_3) \times p_4 \times \left(\frac{(t - p_3)^{p_1 - 1} \times p_2^{p_1} \times e^{-p_2 \times (t - p_3)}}{Gamma(p_1)}\right) \tag{1.2}$$

[8] *fminsearch* function MATLAB documentation
[9] *fminunc* function MATLAB documentation
[10] *fmincon* function MATLAB documentation
[11] Simulated Annealing function *simulannealbnd* MATLAB documentation

- **2-Inverse Logit**

$$p_5 \times \frac{1}{1 + e^{-\frac{t-p_1}{p_3}}} - p_6 \times \frac{1}{1 + e^{-\frac{t-p_2}{p_4}}} \tag{1.3}$$

The user can optimize its own function either by typing directly in the right box (see Figure 10, choose **Personalized Function** in the **Function** dropdown list) or by making it a pre-registered function, as described below. Either way, the function has to follow some ground rules to be interpreted by *Iliski*:

- The function follows MATLAB writing rules and calls MATLAB functions when needed (e.g. the exponential function `exp`). Note that the function can call any function recognized by MATLAB.
- The function is written as an anonymous function, according to the pattern below. Note that `t` is necessarily the x vector and the last parameter.

```
@(Par1, Par2, ..., t)FunctionHandle
```

- As `t` is the x vector, the function handle has to be adapted to it. It may have to use element-wise operator like `./` instead of the classical `/`.

If you want to introduce your function as a pre-registered one, the following rules also apply:

- You have to add your function to the `DefaultParametricFunctions_Iliski.txt` file as a new line.
- The line is made of three parts, each seperated by the ’:’ symbol:
  1. the function name, showed in the GUI ;
  2. the anonymous function handle, as described above ;
  3. the default parameters to be showed in the tab of the GUI (see Figure 10)

As an example, here are the lines for the two-pregistered functions (with # when there is a new line for reading purposes) in `DefaultParametricFunctions_Iliski.txt`:

```
1-Gamma: @(p1, p2, p3, p4, t)((t-p3)>=0).*p4.*(t-p3).^(p1-1).*(p2^p1).* #
       exp(-p2.*(t-p3))./(gamma(p1)):[6 1 0 1]
2-Inverse Logit: @(p1, p2, p3, p4, p5, p6, t)p5*(1./(1+exp(-((t-p1)/p3))))  #
       - p6*(1./(1+exp(-((t-p2)/p4)))):[2 2 2 2 2 2]
```

3.2.3 Parameters for TF computation

Parameters are outlined in Figure 10, although depending on the algorithm some parameters are not used.

First of all, the user can choose the function to use in the corresponding drop-down list. If the user chooses a deconvolutional approach, i.e. **Fourier** or **Toeplitz**, all other computation parameters will turn gray as they are useless. Otherwise, the following parameters are used:

- **Duration**: Duration, in seconds, of the parametric TF to optimize.
  For every optimization algorithms, deterministic or or non-deterministic. The TF dimension is user-defined, with setting of the TF duration and 'Sampling Time' parameters that match the original data or can be augmented by non-linear interpolation.

- **Number of runs**: Number of subsequent runs of the *non-deterministic* algorithm with the same **starting parameters**. Note that a counter is available below the TF plot to go over all the optimized TFs, as many as the number of runs.
  Only for non-deterministic algorithms.

- **Algorithm**: as described above. Check the corresponding checkbox to run **Simulated Annealing** before **fminunc**.

- **Parameters tab**: This tab contains 4 columns.
  For every optimization algorithms, deterministic or or non-deterministic.
  1. Parameters name, as it appears in the default functions file or in the personalized function handle ;
  2. Starting value: the optimization algorithm will set the parameters to those values before working its way to better ones ;
  3. Lower and upper bounds: if the algorithm is *constrainable* (**fmincon**, **fminsearch**, **simulated annealing**), these values will restrain the parameters optimization. Otherwise, these parameters will do nothing.

- **GONG**: as the computation can take some time, checking this box will sonorize the end of the computation process.

A click on the **Compute TF** button starts the computation, once all settings are fine.

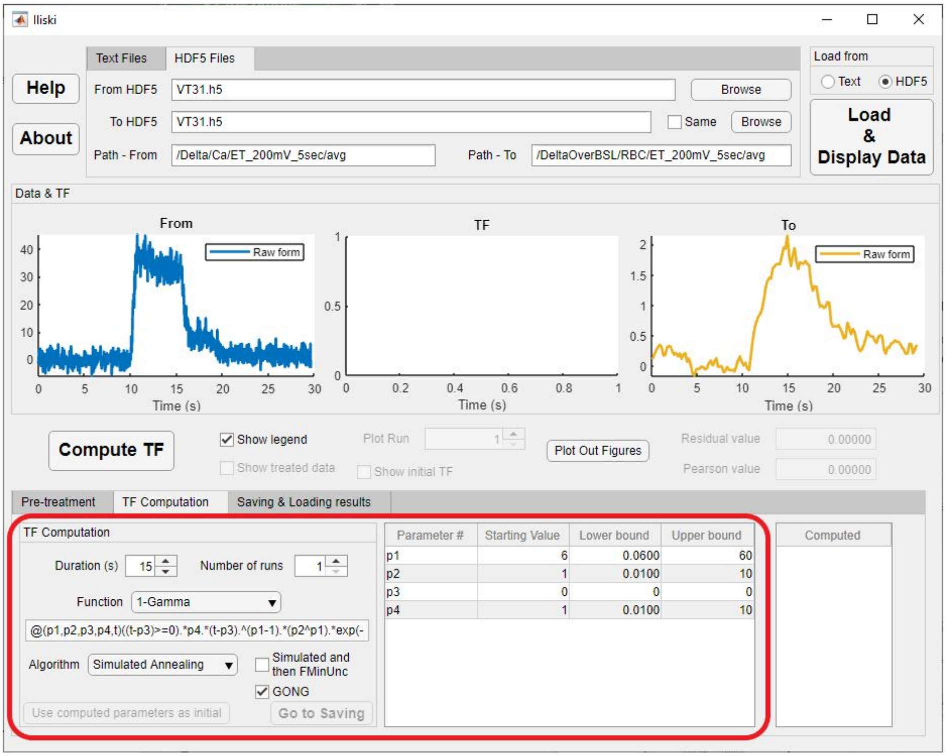

Figure 10: TF computation parameters are circled in red, in the second tab of the lower part of *Iliski*.

3.3 Evaluation of the TF

TFs are evaluated in two different ways, both of which quantify the correspondence between the actual To signal and the prediction made by the TF, i.e. the result of the convolution between From and the TF.

- **Residual sum of squares**: this is the absolute difference between the two timeseries. **This metric is used as the cost function for the optimization algorithm**. It varies from 0 to $+\infty$, 0 being a perfect overlap of the curves[12].

- **Pearson coefficient**: this metric measures how well two timeseries follow each other in their dynamics. It does not take into account the amplitude

[12] Note that this metric only makes sense for a given couple of From & To signals and their pre-processing parameters, since it has no normalization process.

but only the increases and drecreases of the curves. It varies from 0 to 1, 1 being perfect correspondence.

When the user computes multiple runs with a non-deterministic optimization, the runs are ordered by **residual sum of squares** in the increasing order.

3.4 Available options

The table below lists and serves as a memorandum for all the parameters *Iliski* offers. They are divided according to their purposes.

**Input data pre-treatment**	
From	Median filter (number of points)<br>Savitzky-Golay filter (number of points)<br>Sampling time (ms)
To	Median filter (number of points)<br>Savitzky-Golay filter (number of points)
Boxcar function generator	Baseline duration (s)<br>Step duration (s)<br>Total duration (s)<br>Sampling time (s)
**TF computation**	
Function	Fourier deconvolution<br>Toeplitz deconvolution<br>1-Gamma<br>2-Inverse Logit<br>Personalized function
Algorithm[13]	Non-deconvolutional approaches<br>fminunc (D & U)<br>fmincon (D & C)<br>fminsearch (D & C)<br>Simulated Annealing (ND & C)<br>Simulated Annealing and fminunc (ND & U)
Optimization algorithm	TF duration (s, ND & D)<br>Number of runs (#, ND)<br>Starting parameters (a.u., ND & D)<br>Lower and upper bounds (a.u., C)

[13] **D** : Deterministic, **ND** : Non-Deterministic, **U** : Unconstrainable, **C** : Constrainable.

4 Saving a computation and accessing a previous one

Iliski's results can be saved in an Excel file or in MATLAB binary format: MAT-File. Once a computation is done, go to the corresponding tab to save it (see Figure 11). Note that you can only load back results into *Iliski* if they have been saved as MAT-File.

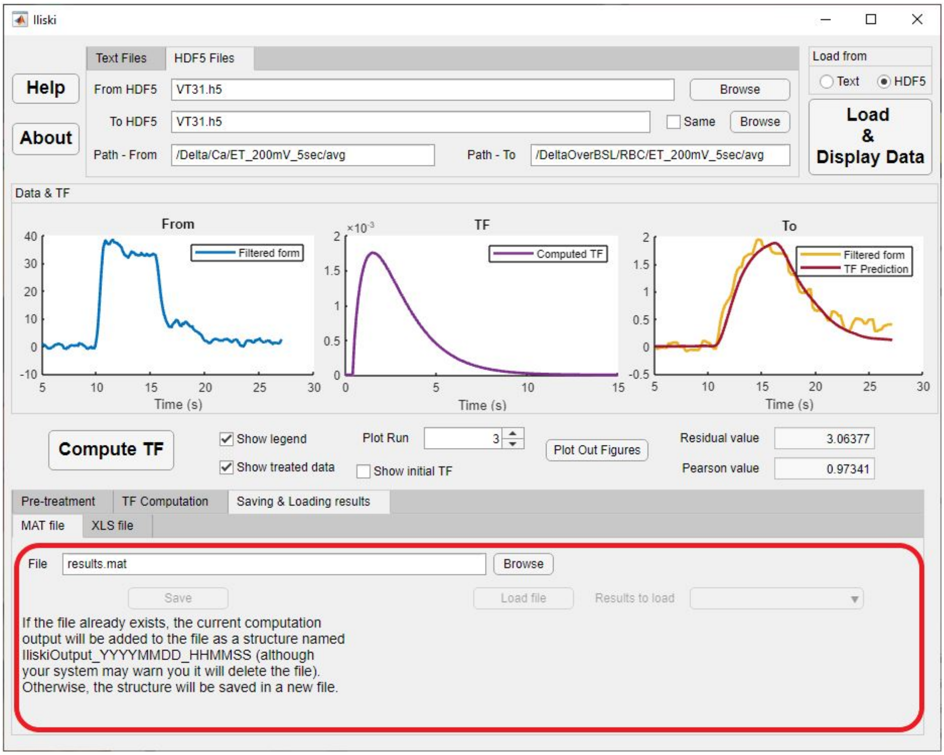

Figure 11: The user can save and load back his results in the third tab of the lower part of *Iliski*, either as a MAT-file or as an Excel Spreadsheet. Here, the displayed tab is for saving as a MAT-file.

4.1 Saving as a MAT-File

If the saving file already exists, it will add the current results to the one already registered inside. Else, it will create the file.

You can load an old result file by clicking on the appropriate button, and go through the results as if they were just computed. If multiple computations were

saved to the same MAT-file, the **Results to load** dropbox will let you choose the one to load.

4.2 Saving as Excel file

Likewise, you can save the results in an Excel file. The tabs in the resulting spreadsheet are quite self-explanatory. Note that if you used a non-deterministic algorithm and multiple runs, although every set of computed parameters will be saved, only the first TF and Prediction timeseries will be written in the corresponding tab (i.e. the one with the smaller residual sum of squares).

5 *Iliski* as a suite of scripts

As described in section 1.3 (page 5), *Iliski* can also be used as scripts. The main function to call is `Iliski_TF.m`, in the `Functions` folder. An example is available in the `test_Ilsiki.m` file, to explain how to use it.

6 Specifications

Iliski has been written with MATLAB 2018a, while the GUI was made with AppDesigner from the same MATLAB version. It has been tested on both Windows 10 and Mac OSX 10.14.3 (Mojave), and it should run on any computer running MATLAB 2018a and the following depencies: Global Optimization Toolbox, Optimization Toolbox and Signal Processing Toolbox. Its standalone version does not need MATLAB to be installed.

7 Contact

In case of questions or bugs, do not hesitate to contact us at ali-kemal.aydin@inserm.fr and davide.boido@cea.fr. An `Errors.log` file inside the software folder is updated with every error *Iliski* encounters, please send the file alongisde your bug description so that we can help you in the best way.

You can also open an issue directly on the GitLab repository, do not hesitate to do so and to thoroughly describe the problem you encounter: https://github.com/alike-aydin/Iliski/issues.

Glossary

from From is the input signal, to be convolved with the Transfer function to get To. 3, 5–12, 17, 18, 21

to To is the output signal, corresponding to the convolution of From and Transfer function. 5–8, 10–12, 17, 18, 21

transfer function Mathematical object representing the relationship between two signals. Hereafter, it is the function linking the input signal From and the output signal To. 1, 5, 21

Acronyms

BOLD-fMRI Blood Oxygen Dependent Level based Functional Magnetic Resonance Imaging. 9, 10

GUI Graphical User Interface. 4, 5, 10, 12, 15

TF Transfer function. 5–7, 10, 12, 16–18, 20, 21

TFs Transfer functions. 4, 13, 16, 17
