# Supplementary material for: Iliski, a software for robust calculation of transfer functions
Source: PLoS Comput Biol. 2021 Jun 14;17(6):e1008614. doi: 10.1371/journal.pcbi.1008614 (PMC8224889; doi:10.1371/journal.pcbi.1008614)
Supplement: Attachment — Submitted filename: Answers to reviewers.pdf [file pcbi.1008614.s005.pdf]

Serge Charpak,
Vision Institute, Sorbonne University
17 rue Moreau, Paris 75012
Telephone: +33 6 62 45 05 89
Email: serge.charpak@inserm.fr

Paris, April 12th, 2021

To the attention of Dina Schneidman, Software Editor

Dear Editor,

We are pleased that our manuscript now entitled: “Iliski, a software for robust calculation of transfer functions”, has been judged suitable for publication in PLOS Computational Biology, providing that we address all the reviewers’ concerns.

Responses to Reviewer #1:

The manuscript presents the software toolbox Iliski; a user-friendly toolbox designed to determine transfer functions between input and output signals.
The determination of transfer functions is elementary and has applications in many fields including biology. Therefore, an easy-to-use program with hints on pitfalls is a great benefit for the community. The program can be operated intuitively. A comprehensive manual is provided. The program reads the provided test data, as well as data from our own lab. Error messages are a little sparse.
The manuscript might benefit, if the authors mention other possible applications more prominently, such as optical impulse response function of biological tissue (DOI: 10.1109/IEMBS.1996.651999) or the testosterone regulation system (ISBN 978-91-554-8857-4). Also in other fields as electronic processing, acoustic or economics transfer functions are elementary. The authors do not compare Iliski to any other program for transfer function determination. Is there really no other program for this purpose in such an elementary field.

We thank the Reviewer for their kind comment and useful advice. We added the proposed references in a new sentence in the introduction at line 74 and list other potential applications outside its usefulness on biological systems (lines 37-41). Regarding the former existence of a similar software, we spent a considerable amount of time looking for a program similar to Iliski without success. We figured out that TF computation being an elementary step in many data analysis, general-purpose coding languages, such as Matlab or Python, have all the necessary tools to work out a TF for each dataset. As the reviewer correctly pointed out, the originality and usefulness of Iliski resides in its interactive environment and the possibility to quickly and easily switch across different approaches for a given dataset. Moreover, direct deconvolution and fitting of functions are 2 different classes of problems and Iliski brings both methods in the same framework. We made these concepts clear in the Design and Implementation section.

Specific points:
line 18: The term ‚biological event‘ is not appropriately used. The point is to link related inputs and

outputs. An event describes only one time point. Furthermore, the toolbox can be used for all kinds of systems and not only in biology.

We removed ‘biological event’ from the manuscript, replacing it with ‘biological processes’ or ‘cellular mechanisms’, appropriately to the context. Regarding the potential uses of Iliski, apart from the modifications made in the Introduction, we agree on its versatility. However, the focus of the Journal is on Biology. To note, Biology often requires testing several approaches to compute TFs. We added a specific sentence at line 74 in the Introduction to emphasize on the versatility.

line 37: The end of the sentence does not fit the beginning: The TF of neurons is not used for the prediction of complex electrical circuits...

We rephrased the sentence pointed out by the Reviewer to explain the multiple applications of TFs.

line 201: In the text, only Simulated Annealing was presented before. Derivative-free and Quasi-Newton were not explained.

We added a short description of the used algorithms. Since they are all standard approaches implemented in the Optimization Toolbox for Matlab, a more detailed description can be found in the functions (whose names are reported in the manuscript) help pages. Our intention was to choose standard but different algorithms to encompass the main options available to tackle an optimization problem. We clarified our intention in the manuscript as well, at lines 177-179, 181, 184-185.

line 231 & figure 4: Even though the resulting residuals of animal 2 are not significantly different, a clear difference is described, therefore it is difficult to refer it as a close performance. A figure showing the residuals of animal 2 is missing (analogous to figure 4b). Why are the residuals of animal 2 so much larger than for animal 1? Can the results of both animals (or more) be considered together? Then the groups would be larger (n=5 is very small).

We did not add a panel with the residuals of animal 2 because there was not any significant difference as it was in the case for animal 1. The two sets of residuals cannot be merged because, although the signals from the two mice have been interpolated to the same sampling time, there is still a difference in the noise level, and residual values strongly depend on this data feature (R2 animal 1 (200 and 200+200 runs): 2.76 ± 0.65; R2 animal 2: 8.53 ± 3.5; the two residual sets are different with $p < 0.001$ (two-tailed T-test, n=10)). R2 plot of animal 2 is below.

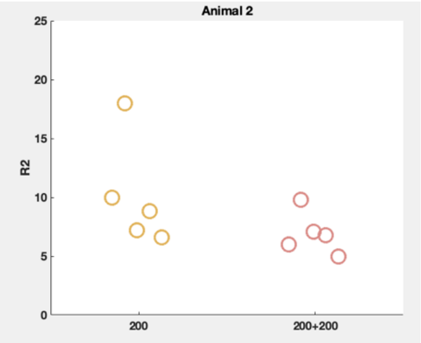

The message of the figure is, indeed, that when we refine the optimization process on our dataset, the main advantage is not a smaller R2, but in the reliability of the TF shape. From another perspective: TFs with very similar R2 can show very different temporal evolution, mainly due to the noise of the data. When choosing an optimization algorithm, one should also take into account the

reliability of the TFs temporal features. We now added the information of the missing panel in the figure legend and made this message clearer in the Results section, lines 249-253.

line 202: How and according to which criteria is a TF classified as biologically inconsistent?

We apologize if the concept of 'biologically consistent' TF was not introduced well enough. We now provide a clear description of what we mean, lines 203-206.

line 212: What is the difference between a run and an iteration?

We thank Reviewer 1 for pointing this omission. As we defined in our previous article (Aydin et al, 2020), we define as a 'run' a single application of the optimization with a given set of initial values, and as a 'iteration' the ensemble of runs sharing the same initial values'. This is now indicated in Results, line 226: '

line 246: The word 'some' overstates: Only the influence of the number of iterations and runs is investigated.

We removed the word to avoid overstatements.

Missing: Demands on the test data: What is the minimum number of measuring points that input and output require for Iliski to run reliably?

We interpreted this question as how many time points are acceptable at minimum, to compute TF. The answer depends on the set parameters, such as the chosen method for TF computation, the eventual selected function and the filtration eventually set. Moreover, the number of points can be artificially augmented by acting on the 'Sampling Time'.

Minor:
line 35: 'addressed' not 'address' Done
figure 5: 'Yes' & 'no' is missing at the box 'are the signals noisy?' Done

<u>Responses to Reviewer #2:</u>

This manuscript that describes use of a relatively simple platform to estimate transfer functions between inputs and outputs. The work is considered interesting in that it provides the public with a platform to estimate simple relationships in data. The platform is well presented and although I did not download and test, I am familiar with this sort of analysis. A few clarifications are requested prior to recommending publication.

Have the authors thought to include square (boxcar) functions as input? This is routinely done in fMRI data analysis. It would be useful to pair this with a different example of output data, especially data with different noise structure. For example, a prior publication from this group has ultrasound imaging data, that would be informative and expand the appeal for this platform.

We really appreciate the feedback provided by Reviewer #2. Iliski does work with boxcar functions as input. In **Fig.1A** the boxcar generator panel is in the bottom-left side of the GUI and, paragraph

2.2 of the manual explains how to use this function. We added a sentence in the Discussion (line 263) and in the Data Loading and pre-treatment (lines 95-96) sections to acknowledge this additional functionality of our SW.

Does the result of the optimization by correlation get a final amplitude adjustment by linear projection (least squares) or is it unit amplitude?

There is no final amplitude adjustment in Iliski, the residual value is thus in unit amplitude. A foot-note page 17 of the User Manual cautions the user on the specificity of this metric.

The authors should state whether this framework is able to handle complex-valued signals (sometimes used to maintain Gaussian noise). No need to modify the platform, just state for clarity.

The reviewer is right in pointing this case out. We did not check for this feature, so we now clearly state this limitation at the end of the 'Data loading and pre-treatment' section of the manuscript and in the User Manual.

Please clarify whether the TF dimension is the same as that of the input or it if is augmented by interpolation in some step.

The sampling time of the TF is defined by the mandatory parameter 'Sampling Time', details are in the User Manual. This is now clarified in both the User Manual and the manuscript in the 'TF computation options' paragraph.

Minor
Line45-46, this sentences are confusing since TFs simplify relationships into potentially single relationships. This needs to be clarified or modified.

We rephrased the sentence (lines 46-47), describing the multiple processes as '*molecular cascades mediated by different cell types'* to highlight the idea that NVC is so complex that, at the moment, an integrated model based on physiology is still lacking.

Line195-96, Replace bonds with BOUNDS.

Done

Line164, TF should not be italicized (that would mean variable T times F(t)), make it non-italicized to define it as one variable. It would be generally useful to also list TF(t) as TF(t; p1, p2, p3, p4) to make it clearer that the goal is to fit/manipulate those arguments.

Done
